# Abdominal Mass Secondary to Human Toxocariasis

**DOI:** 10.21699/ajcr.v8i1.490

**Published:** 2017-01-05

**Authors:** Javad Ghoroobi, Leily Mohajerzadeh, Maliheh Khoddami, Alireza Mirshemirani, Naser Sadeghian, Alireza Mahdavi, Sayeh Hatefi

**Affiliations:** 1 Pediatric Surgery Research Center, Shahid Beheshti University of Medical Sciences, Tehran, Iran.; 2 Pediatric Pathology Research Center, Shahid Beheshti University of Medical Sciences, Tehran, Iran

**Keywords:** Toxocariasis, Human, Abdominal mass

## Abstract

Toxocariasis is an extensive helminthic infection that leads to visceral larva migrans in humans. A 2.5-year-old girl referred for abdominal mass. She had history of pharyngitis for two weeks. There were no other symptoms. Abdominal examination revealed an irregular solid mass in right lower quadrant (RLQ). Abdominal ultrasonography revealed an echohetrogenic large mass in RLQ, liver, and retroperitoneal area. Abdominal CT scan showed a huge mass. At laparotomy a large retroperitoneal mass that involved right liver lobe, bladder, ileocecal valve, small and large intestines was found. At histopathology diagnosis of toxocariasis was made.

## INTRODUCTION

Toxocariasis is a human illness resulting from the dog roundworm (Toxocaracanis), the cat roundworm (Toxocaracati) or the fox (Toxocaracanis). Toxocariasis is often recognized as visceral larva migrans (VLM).[1,2] Fever, cough, wheezing, seizures, rashes, lymphadenopathy, and visual symptoms including decreased vision or blindness are its main features.[3,4] We report a case of human toxocariasis presenting with abdominal mass which was suspected as a malignancy.

## CASE REPORT

A 2.5-year old girl was admitted due to an abdominal mass. She had history of pharyngitis for the last two weeks. She had no other symptoms. At physical examination an irregular solid mass was noted in RLQ approximate 8cm x11cm in size. Laboratory tests showed hemoglobin 10.1gm dl, with leukocytosis and 4% eosinophil. A 24-hour urine for catecholamine level was normal. LDH was 395. Abdominal ultrasonography revealed an echo-heterogenic retroperitoneal mass. CT scan showed a huge mass with mesenteric involvement. Laparotomy showed massive retroperitoneal mass involving right lobe of the liver, bladder, ileocecal valve, small and large intestines. About 50cm of small intestine, cecum, appendix, ascending colon, some mesenteric lymph nodes were removed; ileostomy and colonic mucous fistula were formed. Liver and bladder biopsies were also taken (Fig.1). On microscopy, a diffuse severe chronic inflammation with multiple granulomas, including numerous eosinophils, many lymphocytes and foreign body type giant cells surrounding degenerated worm-like structures found (Fig. 2,3). These structures were also present in some of the foreign body type giant cells. Appendix revealed increased lymphoid follicular hyperplasia. Small intestinal wall showed acute serositis. The resected lymph nodes showed reactive changes. No evidence of malignancy was identified. The final diagnosis was severe chronic and granulomatous inflammation with degenerated structures, suggestive of parasitic infection. Serological test was positive for toxocara IgG. The patient was managed with anti-helminthic therapy. At 2-year follow-up, she has undergone ileostomy reversal and is without any mass or features of the disease.

**Figure:**
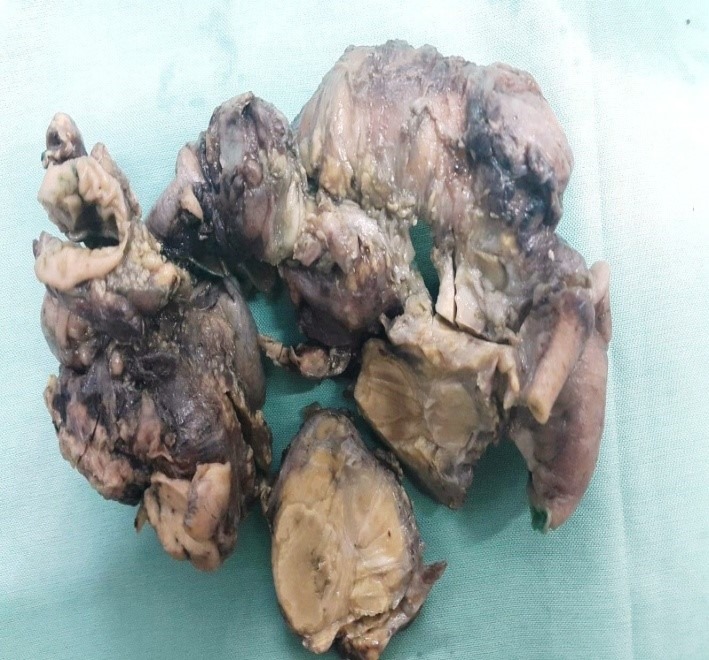
Figure 1: Three encapsulated masses with tan-yellow nodular cut surfaces in the mesocolon.

**Figure:**
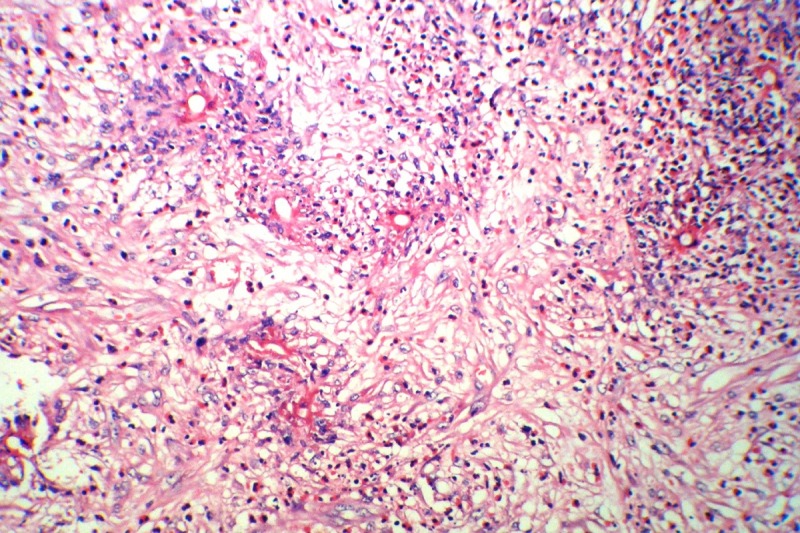
Figure 2: Microscopic examination: Severe chronic inflammation, including numerous eosinophils, many lymphocytes, and foreign body type giant cells containing degenerated worm-like structures. H/E, X250.

**Figure:**
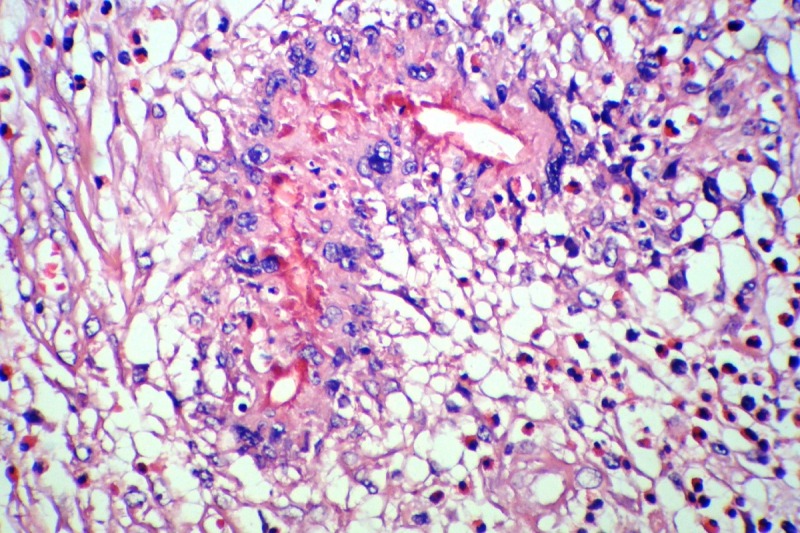
Figure 3: Microscopic examination: Degenerated worm-like structures. H/E, X400.

## DISCUSSION

Population surveys in many countries between healthy group discovered that subclinical toxocariasis is not uncommon.[2,5,6] Toxocaracanis and Toxocaracati are probably the common gastrointestinal worms (helminthes) of domestic dogs and cats and foxes. There are 3 major syndromes: visceral larva migrans (VLM), that engages major organs; covert toxocariasis, which is a milder form of VLM; and ocular larva migrans (OLM), in which eye and optic nerve are involved.[3,4] The person is infected by consuming embryonated eggs (a fully developed larva) from infected vegetables.[1] The diagnosis is usually formed after detection of Toxocara larvae.[1,2,4]. However, serological tests such as PCR, ELISA, frequently employ to identify Toxocara infection.[2,3] This worm produces granulomatous reaction in the viscera and these granulomas can be recognized by ultrasound, MRI, and CT scan as mass abdomen. Toxocariasis often heal itself, because the Toxocara larvae cannot grow-up within human hosts. Either albendazole (preferred) or mebendazole (second line therapy) may be administered.[4] Steroids can be used in case of serositis.

In our case, parasite infection, particularly VLM, had to be differentiated because visceral involvement with eosinophilia and multiple lesion on imaging were observed. We were suspecting it a neuroblastoma but exact diagnosis was guided by histopathology.

## Footnotes

**Source of Support:** Nil

**Conflict of Interest:** None declared

